# Correlation of anti-fungal susceptibility with clinical outcomes in patients with cryptococcal meningitis

**DOI:** 10.1186/1471-2334-12-361

**Published:** 2012-12-20

**Authors:** Chen-Hsiang Lee, Tzu-Yao Chang, Jien-Wei Liu, Fang-Ju Chen, Chun-Chih Chien, Ya-Fen Tang, Cheng-Hsien Lu

**Affiliations:** 1 Division of Infectious Diseases, Department of Internal Medicine, Chang Gung University College of Medicine, Kaohsiung, Taiwan; 2 Department of Laboratory Medicine, Kaohsiung, Taiwan; 3 Department of Neurology, Kaohsiung Chang Gung Memorial Hospital, Chang Gung University College of Medicine, 123 Ta-Pei Road, Kaohsiung, Niao-Sung District, 833, Taiwan; 4 Department of Biological Science, National Sun Yat-Sen University, Kaohsiung, Taiwan

**Keywords:** Cryptococcal meningitis, Fluconazole, Outcome, Susceptibility

## Abstract

**Background:**

This study aimed to investigate the correlation of minimum inhibiting concentrations (MICs), obtained by broth micro-dilution, and clinical response in patients with cryptococcal meningitis.

**Methods:**

Using retrospective analyses covering the period 2001–2010, factors affecting clinical therapeutic cure in patients with cryptococcal meningitis 10 weeks after the start of anti-fungal therapy were identified. Specific emphasis was placed on the role of anti-fungal susceptibility.

**Results:**

Of 46 patients with cryptococcal meningitis identified, 21 were cured after 10 weeks of treatment. Overall, 12 strains (26.1%) were resistant to fluconazole (>8 μg/ml) and 8 (17.4%) had an MIC >1 μg/ml for amphotericin B. Twenty-three patients received combination amphotericin B and fluconazole as their initial antifungal therapy, 17 were given amphotericin B only, five received fluconazole only, and one received a combination of amphotericin B and flucytosine. After 2 weeks, all patients received fluconazole (400–600 mg daily for 8 weeks at least, then 200 mg daily thereafter). The presence of isolates resistant to fluconazole (MIC >8 μg/ml; 4.8% vs. 44%, *p* < 0.01) were statistically significant among patients who were cured. Anti-fungal susceptibility, reflected by fluconazole MIC >8 μg/ml, was an independent predictor of therapeutic cure at 10-week evaluation (OR = 15.7; 95% CI: 1.8-135.9; *p* = 0.01), but higher MIC of amphotericin B (>1 μg/ml) was not.

**Conclusions:**

The MICs of fluconazole, determined by the CLSI method, may be a potential predictor of therapeutic cure in patients with cryptococcal meningitis.

## Background

*Cryptococcus neoformans* is an encapsulated yeast responsible for life-threatening infections, particularly in immuno-compromised patients like those with AIDS [1]. Pharmacologic management usually consists of primary therapy with amphotericin B, with or without flucytosine, followed by maintenance therapy [2]. High rates of fungal persistence and frequent relapse have sparked growing concern among clinicians on the possible emergence of anti-fungal resistance. There are now several published reports of resistance to amphotericin B, fluconazole, or flucytosine in *C*. *neoformans* during treatment [3-6]. Most cases involve resistance to fluconazole in the setting of meningitis in AIDS patients after prolonged treatment or prophylaxis [4-6].

Physicians have long sought a laboratory test to predict clinical outcomes in patients with cryptococcosis. The Clinical and Laboratory Standards Institute (CLSI) recently established a reproducible method for testing the susceptibility of *C. neoformans* to anti-fungal drugs [7]. However, *in vitro* susceptibility testing methods (including the CLSI method and breakpoints) for *Cryptococcus* species have not been well-validated [2]. One report concludes that *in vitro* anti-fungal susceptibility does not predict clinical outcomes in patients with cryptococcosis [8], whereas others report that high minimum inhibiting concentrations (MICs) of fluconazole affect outcomes [9,10]. Unfortunately, these studies are confounded by selection bias because most of them enrolled AIDS patients and most *C. neoformans* isolates are susceptible to fluconazole [8-10].

The present study investigated the correlation between the MICs of amphotericin B or fluconazole (obtained by the broth micro-dilution method described in the CLSI document M27-A3) with the clinical responses of cryptococcal meningitis patients, excluding those with AIDS only. In the context of recent emerging non-susceptible strains of *C. neoformans*, the objective was to validate an *in vitro* measure that could reliably predict patient response after 10 weeks of initial anti-fungal treatment.

## Methods

### Patients and setting

Data covering the period January 2001 to December 2010 were retrospectively collected from patients with cerebrospinal fluid (CSF) cultures positive for *Cryptococcus neoformans* admitted to Kaohsiung Chang Gung Memorial Hospital (KCGMH), a 2300-bed facility serving as a primary care and tertiary referral center in southern Taiwan. If a patient had multiple positive results for *C. neoformans*, only the first instance was included. Demographic and clinical information, including the anti-fungal treatment used in each case, were obtained. The Chang Gung Memorial Hospital’s Institutional Review Board approved the study [No.99-1408C].

Data on clinical variables, including age, sex, underlying diseases (i.e., AIDS, steroids usage, diabetes mellitus, liver cirrhosis, malignancy, chronic renal failure, chronic obstructive pulmonary disease, and organ transplantation) and use of systemic anti-fungal therapy one month prior to first positive cryptococcal CSF culture were collected. Diabetes mellitus was defined as hemoglobin A1c >7%, with random blood glucose level >200 mg/dl or fasting blood glucose level > 126 mg/dl in repeated tests during the hospital stay, or if the patient had previously been diagnosed with diabetes mellitus.

The severity of illness at the time of CSF sampling was assessed using a modified APACHE II scoring method [11], and stratified based on the need for admission to an intensive care unit (ICU). The APACHE II scoring was modified as follows: zero points were given to the items PaO_2_ and pH if arterial blood gas analysis was not performed because of the absence of respiratory distress. Increasing intra-cranial pressure (IICP) was defined as an elevated opening pressure >200 mmH_2_0. If there was evidence of high intracranial pressure with obstruction that needed decompression, drainage was done via ventriculo-peritoneal shunt in accordance with IDSA guidelines [12]. Laboratory data collected included lumbar puncture findings (opening pressure, India ink smear, and CSF levels of cryptococcal antigen), initial serum level of cryptococcal antigen, and results of blood fungal cultures.

Forty-six patients with cryptococcal meningitis were identified during the 10-year study period, including 32 men and 14 women, with age range between 18 and 85 years. Forty-two (91.3%) were tested for HIV and six were infected.

### Clinical evaluation

The 46 patients were divided into 4 groups based on their initial anti-fungal treatment regimen used during the first 2 weeks. Twenty-three patients received a combination of amphotericin B and fluconazole as their initial anti-fungal therapy; 17 had amphotericin B only; 5 received fluconazole (400–800 mg daily) only; and 1 received a combination of amphotericin B and flucytosine. After 2 weeks, patients in all groups received fluconazole (400–600 mg daily for 8 weeks at least, then 200 mg daily thereafter). Follow-up was conducted after 2 weeks of treatment to assess the early outcomes of the disease.

Due to the sub-acute nature of the infection, the primary outcome measure was the clinical outcome after 10 weeks of treatment. Therapeutic cure was defined as improvement or disappearance of symptoms, negative clinical findings (i.e., fever, meningitis, neurological disorder, and abnormal mental status) and sterile CSF culture at the time of evaluation. Therapeutic failure was defined as either 1) clinical deterioration during hospitalization, including progressive hydrocephalus from comparison with initial cranial computed tomography (CT) study; new onset CNS vasculitis demonstrated by brain CT; seizure or status epilepticus with progressive disturbed conscious state; and a modified Barthel Index score <12 [13]; or 2) presence of *C. neoformans* in the patient’s CSF at the time of evaluation. Death resulting from any cause was recorded at the time of evaluation.

### Fungal strain

The processing of specimens and isolate identification were performed by conventional methods and with the YBC identification card (bioMerieux, Marcy l’Etoile, France). The isolated strains were preserved at −70°C until the experiments.

### Isolation of genomic DNA and PCR amplification

Each strain was grown on SDA plates at 35°C for 2 days. Cells were collected and suspended in a TE buffer (100 mM Tris–HCl; pH 8.0, 1 mM EDTA) containing lyticase (Sigma, St. Louis, MO, USA). Glass beads (Sigma) were then added to the micro-tubes, and the samples were incubated at 37°C for 4 h, and mixed in an end-over-end mixer to digest cell walls. Genomic DNA was extracted from the cells with the High Pure PCR template preparation kit (Roche Applied Science, Mannheim, Germany) according to the manufacturer’s instructions.

The serotype of *Cryptococcus neoformans* was identified by multiplex PCR [14]. Briefly, four primers for cloning LAC116 and 2 for CAP64 were used. The amplified products were electrophoretically separated and stained with ethidium bromide. The DNA bands were extracted using a gel extraction kit (QIAquick, QIAGEN Sciences, Germantown, MD, USA) and sequenced directly using a BigDye Terminator Cycle-sequencing kit (ABI PRISM 310NT Genomic Analyzer, Perkin-Elmer Applied Biosystems, Foster, CA, USA).

### Anti-fungal susceptibility testing

Anti-fungal susceptibilities were determined using the broth micro-dilution method according to the CLSI M27-A3 methodology [7]. The anti-fungal agents tested were fluconazole (Pfizer, New York, NY, USA), voriconazole (Pfizer), and amphotericin B deoxycholate (Sigma). Stock solutions were prepared in dimethyl sulfoxide (voriconazole and amphotericin B) or water (fluconazole), and were further diluted in RPMI 1640 medium (Sigma) buffered to a pH level of 7 with 0.165 M 3-(*N*-morpholino) propanesulfonic acid buffer (Sigma). Aliquots of each agent (0.1 ml) at 2× concentrations were dispensed into 96-well micro-dilution trays. Inocula containing 0.5 × 10^3^ to 2.5 × 10^3^ cells/ml were then added to each well and the trays were incubated at 35°C. The final anti-fungal concentrations ranged from 0.06 to 8 μg/ml for voriconazole and amphotericin B, and from 0.25 to 64 μg/ml for fluconazole.

The MICs for voriconazole and fluconazole were read as concentrations causing a 50% reduction in turbidity compared to the growth of the control at 72 h. For amphotericin B, the MICs were read as the concentrations resulting in 100% inhibition relative to that of the growth of controls. *Candida krusei* ATCC 6258 and *Candida parapsilosis* ATCC 22019 were used as quality controls. The interpretative criteria for susceptibility to fluconazole was as published by the CLSI as ≤8 μg/ml [7]. Because there were no established interpretative criteria for amphotericin B and voriconazole, isolates with MICs that were both ≤1 μg/ml were considered susceptible, as suggested by Nguyen and Yu [15] and Lozano-Chiu et al. [16].

### Statistical analysis

Statistical analysis was conducted using the SPSS statistical analysis system. If 2 or more *C. neoformans* strains were isolated from a patient, the first isolated strain was included in the analysis. When *C. neoformans* was isolated from a patient’s blood and CSF, the isolate from the CSF specimen was included in the analysis. Forty-six *C. neoformans* isolates were analyzed. In the univariate analysis, a comparison of contingency data was performed using the Chi-squared test or Fisher’s exact test, as appropriate. Factors with *p* < 0.1 in univariate analyses were separately entered into a multiple logistic regression model to identify independent predictive factors for the successful cure of cryptococcal meningitis at the time of evaluation. Variables with a two-tailed *p* < 0.05 were considered statistically significant.

## Results

The serotype of *C. neoformans* was serotype A in 41 isolates and serotype B in 5 isolates. The susceptibilities of the 46 strains to fluconazole, amphotericin B, and voriconazole were determined by CLSI (Table 1). The widest ranges and highest MICs were for fluconazole (2–32 μg/ml), whereas the lowest MIC was for voriconazole (≤0.5 μg/ml). Overall, 12 strains (26.1%) were not susceptible to fluconazole (>8 μg/ml) and 8 (17.4%) had an MIC >1 μg/ml for amphotericin B.

**Table 1:** **Inhibitory activities of amphotericin B, fluconazole, and voriconazole against clinical isolates of*Cryptococcus neoformans*(n = 46) from initial cerebro-spinal fluid culture**

**Anti-fungal agent**	**Cumulative % inhibited at MIC (μg/ml)**									
	**0.06**	**0.12**	**0.25**	**0.5**	**1**	**2**	**4**	**8**	**16**	**32**
Amphotericin B	0	0	6.5	36.9	82.6	100				
Fluconazole			0	0	0	8.7	28.3	74	95.7	100
Voriconazole	8.7	52.2	91.3	100						

Fifteen patients exhibited worsening symptoms of cryptococcal meningitis and five died from cryptococcal infection on or before the end of the 2-week therapy. Compared to the 20 patients who were not cured, the 26 patients who were cured after 2 weeks of initial therapy showed some statistically significant characteristics, including age <60 years (76.9% of cured patients versus 45% of uncured patients were younger than 60 years; *p* = 0.04). There was no relationship between anti-fungal susceptibility or induction treatment modality and therapeutic cure at the two-week interim analysis (Table 2). Multivariate logistic regression analysis indicated that patient age >60 years (OR = 4.1; 95% CI: 1.1-14.5; *p* = 0.03) was an independent predictive factor for poor clinical outcome (failure and death) of cryptococcal meningitis at the two-week interim evaluation.

**Table 2:** Demographic characteristics, laboratory results, and treatment modalities of patients with cryptococcal meningitis (n = 46) at two-week evaluation

***Variables***	***Cured (%) (n = 26)***	***Not cured***^***#***^***(%) (n = 20)***	***p value***
***Demographics***			
*Male sex*	*19 (73.1)*	*13 (65)*	*0.75*
*Age >60 years*	*6 (23.1)*	*11 (55)*	*0.04*
***Coexisting conditions***			
*AIDS*	*2 (8.3)*^*a*^	*4 (22.2)*^*b*^	*0.38*
*Solid tumor malignancy*	*2 (7.7)*	*4 (20)*	*0.38*
*Hematologic malignancy*	*3 (11.5)*	*1 (5)*	*0.62*
*Steroid usage*	*3 (11.5)*	*5 (25)*	*0.27*
*Liver cirrhosis*	*1 (3.8)*	*4 (20)*	*0.37*
*Chronic renal failure*	*2 (7.6)*	*1 (5)*	*1*
*Diabetic mellitus*	*8 (30.8)*	*4 (20)*	*0.51*
*COPD*	*4 (15.4)*	*0*	*0.12*
*Organ transplantation*	*1 (3.8)*	*0*	*1*
*No known predisposing factor*^*c*^	*6 (23.1)*	*2 (10)*	*0.44*
*Previous anti-fungal therapy*^*d*^	*3 (11.5)*	*4 (20)*	*0.68*
***Severity status***			
*APACHE-II score*	*8.2 ± 2.4*	*10.4 ± 3.7*	*0.19*
*APACHE-II score ≥ 15*	*5 (19.2)*	*5 (25)*	*0.73*
*Shock*	*1 (3.8)*	*0*	*1*
*IICP*	*23 (88.5)*	*18 (90)*	*1*
*ICU admission*	*14 (53.8)*	*8 (40)*	*0.39*
***Initial laboratory data***			
*India ink smear positive*	*12 (46.2)*	*11 (55)*	*0.77*
*CSF opening pressure (mmH*_*2*_*0)*	*248 ± 132*	*252 ± 136*	*0.58*
*CSF WBC count (/μL)*	*32.2 ± 40.6*	*28.8 ± 38.6*	*0.28*
*CSF CAT ≥ 1:1024*	*13 (50)*	*14 (70)*	*0.23*
*Serum CAT ≥ 1:1024*	*9 (34.6)*	*9 (45)*	*0.55*
*Concurrent cryptococcemia*	*6 (23.1)*	*6 (30)*	*0.74*
*Serotype B Cryptococcus neoformans isolate*	*4 (15.4)*	*1 (5)*	*0.37*
*Isolate resistant to fluconazole (MIC >8 μg/ml)*	*4 (15.4)*	*8 (40)*	*0.09*
*Isolate resistant to amphotericin B (MIC >1 μg/ml)*	*3 (11.5)*	*5 (25)*	*0.27*
***Treatment modality in induction therapy***			
*Amphotericin B plus flucytosine*	*0*	*1 (5)*	*0.44*
*Amphotericin B plus fluconazole*	*10 (38.5)*	*13 (65)*	*0.14*
*Amphotericin B alone*	*12 (46.2)*	*5 (25)*	*0.22*
*Fluconazole alone*	*4 (15.4)*	*1 (5)*	*0.37*

Abbreviations: *AIDS* acquired immuno-deficiency syndrome, *CAT* cryptococcal-antigen titer, *COPD* chronic obstructive pulmonary disease, *CSF* cerebro-spinal fluid, *ICU* intensive care unit, *IICP* increasing intra-cranial pressure.

^#^ Non-cured patients included 15 patients who failed to respond to cryptococcal meningitis treatment by the end of two weeks of initial anti-fungal therapy, and five who died from cryptococcal infection before the end of two weeks of initial anti-fungal therapy.

^a^Number of patients available for analysis, n = 24.

^b^Number of patients available for analysis, n = 18.

^c^All 8 patients without known predisposing factor were tested for HIV and none was infected.

^d^Received systemic anti-fungal agents one month prior to first positive cryptococcal cerebro-spinal fluid culture.

*Multivariate logistic regression analysis indicated that age >60 years (OR = 4.1; 95% CI: 1.1-14.5; *p* = 0.03) was an independent predictive factor for poor clinical outcome (failure and death) of cryptococcal meningitis at 2-week evaluation.

Seventeen patients exhibited worsening symptoms of cryptococcal meningitis, and 8 died from cryptococcal infection on or before the end of the 10-week therapy. Compared to the 25 patients who were not cured, the 21 patients who were cured after 10 weeks of initial therapy showed the presence of isolates resistant to fluconazole (MIC >8 μg/ml) was significantly associated with non-cure (4.8% of cured patients versus 44% of uncured patients had resistant isolates; *p* < 0.01) (Table 3). There was no relationship between initial induction treatment modality and therapeutic cure at the 10-week evaluation. Multivariate logistic regression analysis indicated that the presence of isolates resistant to fluconazole (OR = 15.7; 95% CI: 1.8-135.9; *p* = 0.01) was an independent predictive factor of poor clinical outcome (failure and death) of cryptococcal meningitis at the 10-week evaluation.

**Table 3:** Demographic characteristics, laboratory results, and initial treatment modalities of patients with cryptococcal meningitis (n = 46) at 10-week evaluation

***Variables***	***Cured (%) (n = 21)***	***Not cured***^***#***^***(%) (n = 25)***	***p-value***
***Demographics***			
*Male sex*	*15 (71.4)*	*17 (68)*	*1*
*Age >60 years*	*6 (28.6)*	*11 (44)*	*0.36*
***Co-existing conditions***			
*AIDS*	*1 (5.3)*^*a*^	*5 (21.7)*^*b*^	*0.19*
*Solid tumor malignancy*	*2 (9.5)*	*4 (16)*	*0.67*
*Hematologic malignancy*	*3 (14.3)*	*1 (4)*	*0.32*
*Steroid usage*	*2 (9.5)*	*6 (24)*	*0.26*
*Liver cirrhosis*	*1 (4.8)*	*4 (16)*	*0.36*
*Chronic renal failure*	*1 (4.8)*	*2 (8)*	*1*
*Diabetic mellitus*	*7 (33.3)*	*5 (20)*	*0.34*
*COPD*	*3 (14.3)*	*1 (4)*	*0.32*
*Organ transplantation*	*1 (4.8)*	*0*	*0.46*
*No known predisposing factor*^*c*^	*5 (23.8)*	*3 (12)*	*0.44*
*Previous anti-fungal therapy*^*d*^	*2 (9.5)*	*5 (20)*	*0.43*
***Severity status***			
*APACHE-II score*	*7.2 ±3.4*	*10 ± 2.2*	*0.39*
*APACHE-II score ≥ 15*	*4 (19.1)*	*6 (24)*	*0.74*
*Shock*	*1 (4.8)*	*0*	*0.46*
*IICP*	*19 (90.5)*	*22 (88)*	*1*
*ICU admission*	*12 (57.1)*	*10 (40)*	*0.38*
***Initial laboratory data***			
*India ink smear positive*	*9 (42.9)*	*14 (56)*	*0.55*
*CSF opening pressure (mmH*_*2*_*0)*	*254 ± 116*	*246 ± 122*	*0.68*
*CSF WBC count (/μL)*	*30.4 ± 42.8*	*28.6 ± 32.4*	*0.26*
*CSF CAT ≥ 1:1024*	*11 (52.4)*	*16 (64)*	*0.55*
*Serum CAT ≥ 1:1024*	*8 (38.1)*	*10 (40)*	*1*
*Concurrent cryptococcemia*	*4 (19.1)*	*8 (32)*	*0.50*
*Serotype B Cryptococcus neoformans isolate*	*4 (19.1)*	*1 (4)*	*0.16*
*Isolate resistant to fluconazole (MIC >8 μg/ml)*	*1 (4.8)*	*11 (44)*	<0.01*
*Isolate resistant to amphotericin B (MIC >1 μg/ml)*	*2 (9.5)*	*6 (24)*	*0.26*
***Treatment modality in induction therapy***			
*Amphotericin B plus flucytosine*	*0*	*1 (4)*	*1*
*Amphotericin B plus fluconazole*	*7 (33.3)*	*16 (64)*	*0.08*
*Amphotericin B alone*	*11 (52.4)*	*6 (24)*	*0.07*
*Fluconazole alone*	*3 (14.3)*	*2 (8)*	*0.65*

Abbreviations: *AIDS* acquired immuno-deficiency syndrome, *CAT* cryptococcal-antigen titer, *COPD* chronic obstructive pulmonary disease, *CSF* cerebro-spinal fluid, *ICU* intensive care unit, *IICP* increasing intra-cranial pressure.

^#^Non-cured patients included 17 patients who failed to respond to cryptococcal meningitis treatment by the end of 10 weeks of anti-fungal therapy and eight who died from cryptococcal infection before the end of 10-week anti-fungal therapy.

^a^Number of patients available for analysis, n = 19.

^b^Number of patients available for analysis, n = 23.

^c^All 8 patients without known predisposing factor were tested for HIV and none was infected.

^d^Received systemic antifungal agents one month prior to first positive cryptococcal cerebro-spinal fluid culture.

*Multivariate logistic regression analysis indicated that the presence of Cryptococcus neoformans isolates resistant to fluconazole (OR = 15.7; 95% CI: 1.8-135.9; p = 0.01) was an independent predictive factor for poor clinical outcome (failure and death) of cryptococcal meningitis at 10-week evaluation.

Clinical outcomes, initial induction treatment modality, and their relation to the susceptibility of amphotericin B and fluconazole were shown in Table 4. Among 12 *C. neoformans* isolates with high fluconazole MIC (>8 μg/ml), one patient received combined amphotericin B and flucytosine therapy in the first 2 weeks, but died before the 10-week evaluation. Seven patients received combined amphotericin B and fluconazole therapy in the first 2 weeks, of which only one was cured, five failed to respond, and one died. Three patients who received amphotericin B therapy only in the first 2 weeks all failed to respond. One patient who received fluconazole therapy alone in the first 2 weeks died at the 10-week evaluation. For eight isolates with high amphotericin B MICs (>1 μg/ml), five patients received combined amphotericin B and fluconazole therapy in the first 2 weeks, but only one was cured and 4 failed to respond at the 10-week evaluation. Two patients received amphotericin B therapy only in the first 2 weeks, and both died before the 10-week evaluation. One patient received fluconazole alone in the first 2 weeks and was cured. For susceptibility to fluconazole, there was a trend for resistance (MIC >8 μg/ml) to be associated with poor clinical outcome (failure and death), irrespective of initial treatment modality, at the 10-week evaluation.

**Table 4:** **Susceptibility testing results (CLSI method) for*Cryptococcus neoformans*isolates from patients, with subsequent clinical outcomes after 10 weeks of antifungal therapy**

***Initial treatment***^***a***^***and outcome***^***b***^	***No of patients/strains***	***No. of strains not susceptible to anti-fungal agent***^***c***^	
		***AMB***	***FCZ***
*AMB + 5FC*			
*Cure*			
*Failure*			
*Death*	*1*		*1*
*AMB + FCZ*			
*Cure*	*7*	*1*	*1*
*Failure*	*12*	*4*	*5*
*Death*	*4*		*1*
*AMB alone*			
*Cure*	*11*		
*Failure*	*4*		*3*
*Death*	*2*	*2*	
*FCZ alone*			
*Cure*	*3*	*1*	
*Failure*	*1*		
*Death*	*1*		*1*

Abbreviations: *5FC* flucytosine, *AMB* amphotericin B, *FCZ* fluconazole.

^a^The 46 patients were divided into 4 groups based on their initial anti-fungal treatment regimen used during the first 2 weeks. Thereafter, all patients received fluconazole (400–600 mg daily for 8 weeks at least then 200 mg daily).

^b^Therapeutic cure was defined as improvement or disappearance of symptoms, negative clinical findings and the cerebro-spinal fluid culture was sterile after 10 weeks of anti-fungal therapy. Therapeutic failure was associated with the persistence of symptoms or cerebro-spinal fluid culture still yielding C. neoformans after 10 weeks of anti-fungal therapy. Death was due to any cause before the end of 10 weeks of anti-fungal therapy.

^c^The interpretative criteria for susceptibility to fluconazole were those published by the CLSI [7]. The criteria for susceptibility to amphotericin B were those proposed by Nguyen and Yu [15].

## Discussion

Cryptococcal meningitis is described as an opportunistic infection in immuno-compromised patients such as people infected with HIV, but it can also affect apparently healthy people [17]. In the present study, only 6 (13%) of the 46 patients identified with cryptococcal meningitis were documented as having AIDS. Sun et al. [18] reported a mortality rate of 20.7% for HIV-infected patients within 10 weeks of hospitalization with cryptococcosis. Similarly, the overall mortality rate in the present study after 10 weeks of initial anti-fungal therapy for cryptococcal meningitis was 17.4% (8 patients died). The similarity in the mortality rates of these two studies suggests that mortality rates caused by cryptococcal meningitis may not be different for HIV-positive and HIV-negative patients.

Factors predictive of poor prognosis and mortality in patients with cryptococcal meningitis include (i) cryptococcus growth from sites other than CSF; (ii) alteration in mental status; (iii) IICP; (iv) CSF glucose <40 mg/dL; (v) CSF cell count <20 cells/mm^3^; (vi) high CSF cryptococcal antigen titer [19]; and (vii) positive India ink smear [20]. The goal of treatment includes both cure of the infection and prevention of long-term CNS system sequelae [2]. Prognostic factors for mortality may be overlooked because death may not be attributed to cryptococcosis itself. Therefore, cure of the infection is the endpoint of outcome evaluation in the current study.

High CSF cryptococcal antigen titer and the isolation of cryptococci from extra-neural sites indicate increased fungal load in patients with cryptococcal meningitis [21]. Although these clinical characteristics do not affect the clinical outcomes used to determine therapeutic cure in the current study, there is a consistent trend wherein patients who failed were more likely to have co-morbidities and that severity of illness that would predispose them to a poorer clinical outcome. The cured patients were younger at 2-week interim analysis (Table 2). Other negative results in this study may have been due to low statistical power, rather than a true lack of association between the clinical characteristic and therapeutic cure.

Differing approaches exist for the *in vitro* measurement of *C. neoformans* susceptibility to anti-fungal agents. Anaissie et al. [22] evaluated YNB, RPMI 1640 medium broth, and Eagle’s minimum essential medium broth, and found that RPMI 1640 broth was a suitable medium to test the susceptibility of *C. neoformans*. Franzot and Hamdan [23] corroborated these findings. RPMI 1640 medium is the standard broth recommended by CLSI [7]. When using this medium, MIC determinations require 72 h of incubation. Accordingly, the current study evaluated *in vitro* activities of *C. neoformans* by the broth micro-dilution method, as described in the CLSI document M27-A3 [7].

For *in vitro* anti-fungal susceptibility testing of *C. neoformans*[7], no breakpoint is currently available for any drug [2] and the role of the susceptibility of the initial isolate as a predictor of both early and late clinical treatment failure remains controversial [8-10]. One study with a sample of 74 patients with cryptococcosis (60 of whom had AIDS) found that *in vitro* anti-fungal susceptibility cannot predict the outcome two weeks after the start of anti-fungal therapy [8]. However, the fluconazole MICs of these isolates, which include *C. neoformans* strains, are all ≤4 μg/ml [8]. Witt et al. explored the utility of MICs as predictors of 2-week treatment failure in patients receiving fluconazole for acute AIDS-associated cryptococcal meningitis, and found that the low MIC of fluconazole was independently associated with successful treatment [9]. Aller et al. also reported that the clinical outcomes of 25 patients (24 with AIDS) after 10 weeks of fluconazole maintenance therapy might be better when the infecting *C. neoformans* strain was inhibited by lower concentrations of fluconazole for eradication (MICs ≤8 μg/ml) than when patients were infected with strains that required higher fluconazole concentrations (MICs >8 μg/ml) [10]. These two studies both report reduced treatment efficacies when the fluconazole MIC is >8 μg/ml [9,10].

As in the present study, the MIC of fluconazole is associated with early clinical outcome due to the very few patients who receive fluconazole alone as induction therapy. The use of combined therapy makes this problematic. Rather than focusing on early outcome, it may make more sense to focus on the 10-week outcome when the patients receive fluconazole as maintenance therapy.

In the present study, only 13% patients had AIDS and strains with higher fluconazole MICs (>8 μg/ml) were specifically investigated. Despite using different testing parameters, an association between high fluconazole MICs (>8 μg/ml) and lack of therapeutic cure is evident. One explanation is that strains with high MIC for fluconazole (≥32 μg/ml) are more virulent than strains that have low MIC for fluconazole (≤8 μg/ml) [24]. To date, two studies has demonstrated a strong correlation between quantitative measures of *in vitro* amphotericin B susceptibility and the quantitative response observed in patients [25,26]. In the current study, the discrepancies in susceptibilities to amphotericin B or fluconazole, and clinical outcomes may be due to the small number of patients infected by strains with high MICs of amphotericin B. Further investigation is necessary on the correlation between amphotericin B susceptibility and outcomes in patients with cryptococcosis.

In a previous study, *C*. *neoformans* were collected from the CSF specimens of patients with cryptococcal meningitis between 1998 and 2002 [27]. By comparison, the current study found that the MIC_90_ of fluconazole had increased from 4 μg/ml [27] to 16 μg/ml for the CSF specimens collected between 2001 and 2010 (Table 1). Non-susceptibility to fluconazole was also an emerging problem, according to surveillance data. This is especially true in certain global regions, notably Africa, where prophylactic fluconazole is prescribed widely and the drug is often used alone as the primary therapy for cryptococcosis [4-6]. Initial treatment modality was not associated with clinical outcome in either univariate or multivariate analysis. Only one patient infected by strains with higher fluconazole MICs (>8 μg/ml) received fluconazole therapy alone in the first 2 weeks, and this patient died before the end of the 2-week evaluation. Only two patients infected by strains with higher amphotericin B MICs (>1 μg/ml) received amphotericin B therapy alone in the first 2 weeks, and both died before the end of the 2-week evaluation. Thus, early mortality (first 2 weeks) from cryptococcal meningitis is more likely elated to severity of disease at presentation than treatment response.

Moreover, 12 patients infected by strains with higher fluconazole MICs (> 8 μg/ml) received fluconazole treatment after 2 weeks initial antifungal therapy, and only one was cured at the end of the 10-week evaluation. This suggests that high fluconazole MIC is associated with 10-week treatment failure when fluconazole is used for maintenance therapy after induction therapy.

This study was subject to certain limitations. First, the sample size was relatively small and made retrospective use of patients’ medical records, which usually meant that some data were missing or misclassified. Thus, the findings may underestimate the “true” independent predictor of outcome in this study. Furthermore, the numbers of variables considered for the multiple logistic regression analysis was small. Based on the stepwise procedures, only one variable was selected as the important variable predicting the outcomes. The maximum likelihood estimates of the coefficients are valid in the analysis. Not all the patients were checked for HIV because two patients died before they could be screened for HIV and another two patients did not accept HIV screening. Second, flucytosine was unavailable at the study institution during the 10-year study period and only one patient received the most rapidly fungicidal regimen (amphotericin B plus flucytosine) [28]. Third, the start of induction anti-fungal therapy was different for each patient according to the preference of his/her doctor, which might cause potential bias in statistical analysis. Lastly, no standardization was conducted for certain aspects of data collection, such as amphotericin B or fluconazole levels in serum and CSF. This limits the ability to delineate the relationship between drug dosage, resulting serum and CSF levels, and treatment outcomes.

## Conclusions

The present study suggests that primary non-susceptibility to amphotericin B and fluconazole is common. The MICs determined at the time of diagnosis of cryptococcal meningitis by the CLSI M27-A3 broth micro-dilution method may potentially predict the likelihood of therapeutic cure. Further large-scale studies are required to corroborate the findings.

## Competing interests

This study was sponsored by Pfizer funded, which was acquired in May 2010. The authors declare that they have no competing interests.

## Authors’ contributions

CHL, JWL and CHL took the initiative to develop the research project. TYC, FJC, CCC, and YFT participated in reviewing the clinical data and analysis. CHL and CHL contributed to the manuscript writing. All authors read and approved the final manuscript.

## Pre-publication history

The pre-publication history for this paper can be accessed here:

http://www.biomedcentral.com/1471-2334/12/361/prepub
